# Genetic Variants in *MUC4* Gene Are Associated with Lung Cancer Risk in a Chinese Population

**DOI:** 10.1371/journal.pone.0077723

**Published:** 2013-10-21

**Authors:** Zili Zhang, Jian Wang, Jianxing He, Zeguang Zheng, Xiansheng Zeng, Chenting Zhang, Jinmei Ye, Yajie Zhang, Nanshan Zhong, Wenju Lu

**Affiliations:** 1 State Key Laboratory of Respiratory Diseases, Guangzhou Institute of Respiratory Disease, The First Affiliated Hospital, Guangzhou Medical University, Guangzhou, Guangdong, China; 2 Department of Respiratory Medicine, Xiangyang Central Hospital, Xiangyang, Hubei, China; 3 Department of Pathology, Guangzhou Medical University, Guangzhou, Guangdong, China; 4 Department of Laboratory Medicine, The First Affiliated Hospital, Guangzhou Medical University, Guangzhou, Guangdong, China; Huazhong University of Science and Technology, China

## Abstract

Mucin MUC4, which is encoded by the *MUC4* gene, plays an important role in epithelial cell proliferation and differentiation. Aberrant *MUC4* overexpression is associated with invasive tumor proliferation and poor outcome in epithelial cancers. Collectively, the existing evidence suggests that *MUC4* has tumor-promoter functions. In this study, we performed a case-control study of 1,048 incident lung cancer cases and 1,048 age- and sex frequency-matched cancer-free controls in a Chinese population to investigate the role of *MUC4* gene polymorphism in lung cancer etiology. We identified nine SNPs that were significantly associated with increased lung cancer risk (*P* = 0.0425 for rs863582, *0.0333* for rs842226, *0.0294* for rs842225, *0.0010* for rs2550236, *0.0149* for rs2688515, *0.0191* for rs 2641773, *0.0058* for rs3096337, *0.0077* for rs859769, and *0.0059* for rs842461 in an additive model). Consistent with these single-locus analysis results, the haplotype analyses revealed an adverse effect of the haplotype “GGC” of rs3096337, rs859769, and rs842461 on lung cancer. Both the haplotype and diplotype “CTGAGC” of rs863582, rs842226, rs2550236, rs842225, and rs2688515 had an adverse effect on lung cancer, which is also consistent with the single-locus analysis. Moreover, we observed statistically significant interactions for rs863582 and rs842461 in heavy smokers. Our results suggest that *MUC4* gene polymorphisms and their interaction with smoking may contribute to lung cancer etiology.

## Introduction

Lung cancer is the most common cancer in the world and accounted for 13% (1.6 million) of total cases and 18% (1.4 million) of cancer deaths in 2008 [1]. In China, the incidence and mortality rates of lung cancer have grown rapidly in the past few decades [2], and it is now the leading cause of cancer mortality; the average 5-year survival rate is <15% [3,4]. The lung cancer epidemic is directly attributable to cigarette smoking, which accounts for 87% of lung cancer cases. However, only a small percentage of smokers (<20%) develop lung cancer in their lifetime [5], suggesting that genetic susceptibility may play a role in lung cancer development.

Exposure to cigarette smoke stimulates an inflammatory cascade in airway epithelial cells For example, tobacco smoke generates reactive oxygen species that could injure the lung epithelium, resulting in altered permeability, goblet cell hyperplasia, as well as recruitment of neutrophils and macrophages to the airway [6–9]. Chronic inflammation causes prolonged irritation and activates local host responses, which ultimately promote cell proliferation [10]. Sustained cell proliferation facilitates tumor formation and progression in an angiogenic environment rich in inflammatory cells, growth factors, and activated stroma [11,12]. It has been demonstrated that one-third of all cancers are preceded by chronic inflammation [13]. Case-control studies have demonstrated an increased risk of lung cancer in patients with inflammatory airway phenotypes, such as asthma, bronchitis, and emphysema [14,15]. Recent data suggest that cigarette smoke activates airway epithelial cells and immune cells to release proinflammatory cytokines, such as cyclooxygenase-2 (cox-2), interleukins-4, 6, and 8 (IL-4, -6, -8) and tumor necrosis factor-α (TNF-α). 

Mucins have long been known to be target molecules of inflammatory reactions, and inflammatory diseases of the epithelium are often characterized by mucin upregulation and hypersecretion [16–20]. Moreover, abnormal *MUC4* expression has been reported in various cancers, such as pancreatic adenocarcinomas [21] and colon carcinomas [22], as well as in other lung and airway inflammatory diseases including cystic fibrosis and chronic obstructive pulmonary disease [23–25]. Growth factors are thought to be involved in mucus-secreting cell production because hypersecretory diseases are associated with abnormal epithelial cell growth and proliferation [26].

In addition to its adverse effects in inflammatory diseases, *MUC4* also plays a critical role in regulating diverse processes in lung stromal/parenchymal cells, including apoptosis and metastasis. *MUC4* acts as an intramembrane ligand for ErbB2/HER2/neu and potentiates its autophosphorylation [27]. It has been found that *MUC4*-induced ErbB2/neu signaling may mediate the antiapoptotic function of *MUC4* [28]. Moreover, *MUC4* may possess a tumor-promotion function, in part by regulating *HER2* gene expression. ErbB2/HER2 expression levels have been correlated with tumor size and lymph node metastasis, suggesting the involvement of ErbB2 and ErbB2-mediated signaling in tumorigenesis [29]. Taken together, these observations imply that *MUC4* may promote tumor progression in human lung cancer pathogenesis.

The present work was motivated by the biological plausibility that genetic variation in *MUC4* could alter its expression level or biochemical function and thus may have an impact on individual lung cancer risk. To test this hypothesis, we conducted a case-control study of 1,048 incident lung cancer cases and 1,048 age- and sex- frequency-matched, cancer-free controls in a Chinese population. We also investigated potential interactions between tagSNPs of the *MUC4* gene and cigarette smoking in lung cancer risk. 

## Methods

### Study subjects

The study design and subject recruitment were described as below: briefly, the 1,048 lung cancer patients and 1,048 cancer-free controls were genetically unrelated ethnic Han Chinese from Guangzhou City. Patients with histopathologically confirmed incident lung cancer were consecutively recruited from September 2009 to September 2011 in the Thoracic Surgery Department of The First Affiliated Hospital of Guangzhou Medical University. The 1,048 cancer-free controls that were frequency matched to patients by sex and age (±5 years) were randomly selected from the Health Examination Center of the same hospital during the same time period. Before recruitment, written informed consent was obtained from each eligible subject, and a structured questionnaire was administered by interviewers to collect information on demographic data and environmental exposure history, including tobacco smoking and alcohol intake. Subjects were identified as nonsmokers or smokers. Individuals who had smoked fewer than 100 cigarettes in their lifetime were defined as nonsmokers; otherwise, they were defined as smokers (those smokers who stopped smoking for >1 year were also defined as smokers). Pack-years were calculated by multiplying the number of packs of cigarettes smoked per day by the number of years the person has smoked. Similarly, participants who had consumed alcoholic beverages at least once a week for the previous year were defined as drinkers, and the others were considered nondrinkers. Family history of cancer was defined as any self-reported cancer in first-degree relatives (parents, siblings, or children). After the interview, a 5-ml venous blood sample was collected from each participant. The study was approved by the institutional review board of Guangzhou Medical University (Ethics Committee of The First Affiliated Hospital: GZMC2009-08-1336).

### Selection of SNPs of MUC4

**Figure 1:**
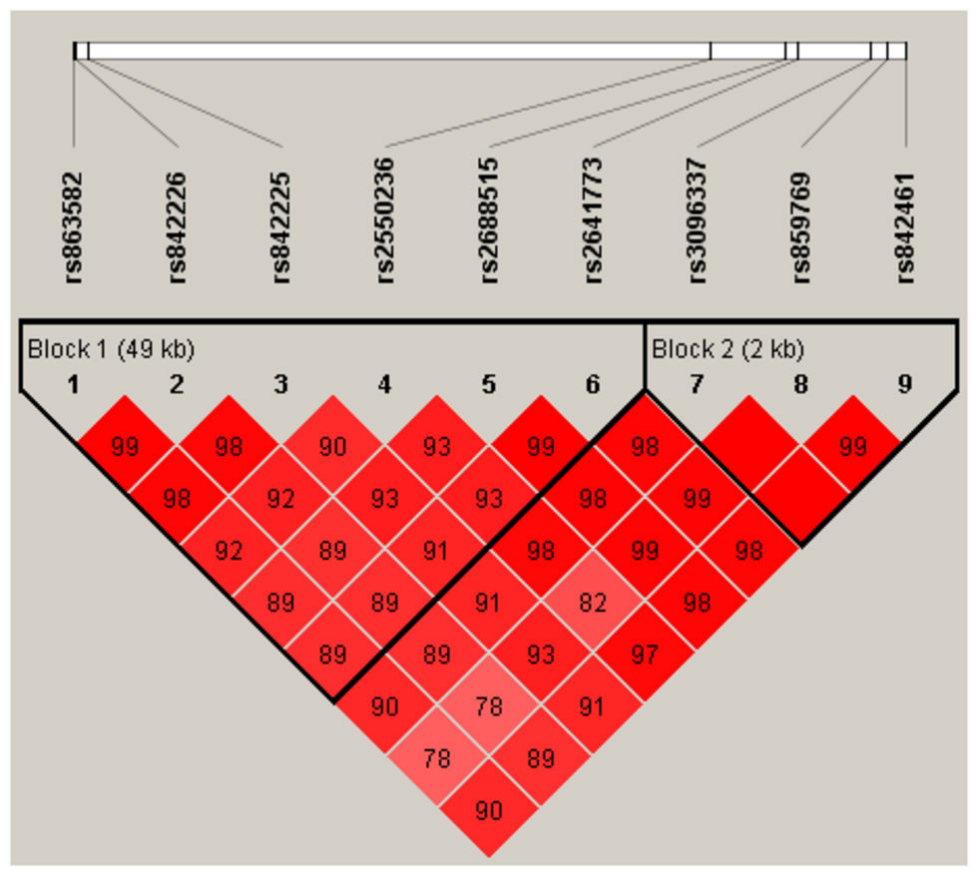
Graphical representation of the SNP locations and LD structure of nine genotyped SNPs of *MUC4* in 1,048 southern Han Chinese controls. The exact SNP positions are listed in Table 1. Two haplotype blocks (colored) were defined by the Haploview program using the approach described by Gabriel et al. ^[34]^ with default settings (the 95% CI for a strong LD was minimal for upper 0.98 and low 0.7, and maximal for a strong recombination of 0.9, and a fraction of strong LD in informative comparisons was at least 0.95). The rs number (top, from right to left) corresponds to the SNP name, and the numbers in squares are D' values (|D'|×100). The measure of LD (D') among all possible pairs of SNPs is shown graphically according to red shading, where white represents very low D', and dark red represents very high D'.

The human *MUC4* gene is ~211 kb in size and is located on chromosome 3 in region q29 [30]. To identify SNPs that were related to lung cancer, we first selected 296 of *MUC4* SNPs with minor allele frequency (MAF) > 5% from both dbSNP (http://www.ncbi.nlm.nih.gov/SNP, accessed 9/9/2012 and HapMap databases [Han Chinese]) (File **S1**), and genotyped them in a small subset of samples from 300 randomly selected pairs of case and control subjects from 1048 pairs on an Illumina high-throughput genotyping platform (Genome Analyzer IIx, Illumina Inc., San Diego, CA). Out of this group, we identified nine SNPs (rs863582, rs842226, rs842225, rs2550236, rs2688515, 2641773, rs3096337, rs859769, and rs842461) that exhibited significant frequency differences between cases and controls (data not shown). Genotype frequencies of SNPs can be influenced by sample sizes [31]. To minimize the bias due to small sample size, we next conducted direct sequencing for the whole set of 1,048 pairs of case and control samples using the ABI PRISM 7500 Sequence Detection System (Applied Biosystems, Foster City, CA) to confirm the above genotyping results (Table **1**). The results from the two platforms were found to be 100% concordant; therefore, we provided association results from the entire set of 1048 pairs in this paper. Finally, we identified two tagSNPs (rs863582 and rs842461) according to the following criteria: a minimal set of haplotypes that ensure an r^2^ of at least 0.8 to cover all possible haplotypes that had a frequency of at least 5% as evaluated by the tagSNPs program [32]. In addition, as shown in Figure **1**, the reconstructed linkage disequilibrium (LD) plot identified two blocks for the above nine SNPs in 1,048 control subjects: block 1 for rs863582, rs842226, rs842225, rs2550236, rs2688515 and rs 2641773; and block 2 for rs3096337, rs859769 and rs842461. Among these SNPs, we found the one in block2 were in high LD with each other (r^2^_min_ > 0.80, D' = 1.00, see Table **S1** for each pair), and therefore we chose rs842461 to represent all three. 

**Table 1:** Genotyped *MUC4* SNPs.

Gene name	NCBI	Chromosome	Inter-marker	Location	Base	MAF			*P* ^c^	*P* ^d^	*P* value	Geno-
and locus	SNP ID	Position^a^	distances	in gene	change						for	typing
			(bp)	region		In Database^b^	Case	Control			HWE^e^	rate (%)
MUC4,	rs863582	195478694	5058	Intron 21	T>C	**0.386**	0.3259	0.2899	*0.0116*	*0.0315*	*0.9022*	99.95
3q29	rs842226	195478861	167	Intron 21	C>T	0.082	0.3228	0.2872	*0.0129*	*0.0372*	*0.8944*	98.81
	rs842225	195479748	887	Intron 21	A>G	0.438	0.3867	0.3474	*0.0091*	*0.0513*	*0.6684*	97.85
	rs2550236	195522321	42573	Intron 1	G>A	0.164	0.3327	0.2974	*0.0152*	*0.1144*	*0.9966*	97.52
	rs2688515	195527471	5150	Intron 1	A>G	0.452	0.3672	0.3244	*0.0036*	*0.0223*	*0.9952*	99.33
	rs2641773	195528226	755	Intron 1	A>C	0.452	0.3716	0.3298	*0.0048*	*0.0171*	*0.9857*	98.90
	rs3096337	195533332	5106	Intron 1	A>G	0.185	0.3087	0.2634	*0.0012*	*0.0081*	*0.6992*	100.0
	rs859769	195534413	1081	Intron 1	T>G	0.415	0.3459	0.3004	*0.0016*	*0.0059*	*0.8740*	99.81
	rs842461	195535614	1201	Intron 1	A>C	0.170	0.3097	0.2643	*0.0012*	*0.0048*	*0.8830*	99.67

^a^ SNP position in NCBI dbSNP (http://www.ncbi.nlm.nih.gov/SNP , accessed 9/9/2012)

^b^ MAF from both HapMap and dbSNP databases, the MAF in bold is from the HapMap database (Han Chinese)

^c^ *P* value for difference in allele distributions between cases and controls

^d^ Generated by 10,000 permutations

^e^ Hardy-Weinberg equilibrium (HWE) *P* value in the control group

### Genotyping assays

The genomic DNA of subject’s blood samples was extracted with a QIAGEN Blood DNA Kit (Venlo, The Netherlands). An allelic discrimination method using allele-specific fluorogenic probes (the 5 nuclease assay with MGB probes and TAMRA probes, as used in the Taqman assay [33] was chosen for genotyping using a ABI PRISM 7500 Sequence Detection System). Primers and probes are described in Table **S2** and were designed by Primer Express 3.0 (Applied Biosystems) and synthesized by Shanghai GeneCore Biotechnologies (Shanghai, China). Polymerase chain reaction (PCR) was performed in 10-μl reaction systems. The PCR protocol consisted of an initial melting step at 95°C for 10 min, 40 cycles of 95°C for 15 s, and 60°C for 1 min. A multicomponent algorithm was used to calculate distinct allele signal contributions from fluorescent measurements for each sample with the ABI 7500HT real-time PCR system. The genotypes were automatically determined by Sequence Detection Systems software 2.3 (Applied BioSystems) (Figure **S1**). In the genotyping assays, 10% samples were randomly selected to perform repeated assays for each SNP, and the results were 100% concordant. 

### Statistical analyses

Two-sided χ^2^ tests were used to assess differences in selected demographic variables, smoking status, pack-years of smoking, family history of cancer, frequencies of *MUC4* alleles, and genotypes between the cases and controls. Goodness-of-fit to Hardy-Weinberg equilibrium (HWE) in controls was also evaluated with a χ^2^-test for each SNP. Akaike’s information criteria (AIC) [34] were applied to select the most parsimonious genetic model for each SNP. Odds ratios (ORs) and corresponding 95% confidence intervals (CIs) were measured with an unconditional logistic regression model with adjustments for age, sex, smoking status, alcohol drinking status, and family history of cancer. Stratification analyses were also performed by variables of interest, such as age, sex, smoking status, alcohol drinking status, family history of cancer, and histologic types. The pairwise LD among the SNPs was calculated using Lewontin’s standardized coefficient D', and LD coefficient r^2^ [35], and haplotype blocks were defined by the method described by Gabriel et al. [36] using publicly available Haploview software (http://www.broad.mit.edu/personal/jcbarret/haplo/) with default settings (the CI for a strong LD was minimal for upper 0.98 and low 0.7, and maximal for a strong recombination of 0.9, and a fraction of strong LD in informative comparisons was at least 0.95). Each common haplotype (MAF > 0.05) was compared between all cases and controls and in each stratum of cumulative smoking dose to determine whether smoking influenced the risk associated with *MUC4* variants by using haplo.stats (available at http://mayoresearch.mayo.edu/mayo/research/schaid_lab/index.cfm). In addition, a PHASE 2.1 Bayesian algorithm [37] was used to validate the haplotype frequencies estimated by Haplo.stats and infer diplotype frequencies based on the observed genotypes. Diplotype (haplotype dosage, an estimate of the number of haplotype copies) was the most probable haplotype pair for each individual. Unconditional logistic regression analyses were used to estimate ORs and 95% CIs for case-control subjects carrying one to two copies versus zero copies of each common haplotype for the dichotomized diplotypes (Table **S3**). The issue of multiple tests was controlled by performing 10,000 permutation tests.

To explore potential interactions between the tagSNPs and smoking status, we performed multiple tests to assess result consistency, including analyses of specific categories of cumulative smoking exposure (i.e., pack-years), genotype-smoking joint-effects, and interaction models that considered both discrete (nonsmokers, light smokers [≤20 pack-years] and heavy smokers [>20 pack-years]) and continuous (square root of pack-years) variables for cumulative smoking exposure. Statistical analyses were performed using SAS 9.2 software (Cary, NC, USA).

## Results

### Study population characteristics

The characteristics of the 1,048 lung cancer patients and 1,048 cancer-free controls are described in Table **S4**. The lung cancer cases and controls were adequately matched for age and sex (*P = 0.7597* and *0.7734*, respectively). Cigarette smoking was associated with increased risk of lung cancer among heavy smokers (OR = 1.66 and 95% CI = 1.37-2.03, data not shown). Among the 1,048 lung cancer cases, 790 (75.38%) were defined as non-small-cell lung cancer (384 adenocarcinoma, 368 squamous cell carcinoma, and 37 large-cell carcinoma), 121 (11.55%) were small-cell lung cancer, and 138 (13.17%) patients had other carcinomas.

### Association between individual SNPs and lung cancer risk

As summarized in Table **1**, the genotype frequency distributions of the nine selected SNPs ((rs863582, rs842226, rs842225, rs2550236, rs2688515, 2641773, rs3096337, rs859769, and rs842461) in control subjects were all consistent with those expect from the HWE model (all *P* > 0.05). One SNP (rs2688515) in this Chinese population represented an MAF that was 12.76% lower than reported in the dbSNP database (http://www.ncbi.nlm.nih.gov/SNP, accessed 9/9/2012), whereas the other SNPs (rs842226) represented an MAF 20.52% higher than those reported in the HapMap SNP database (Han Chinese), which may reflect either a diverse population difference or frequency bias due to small sample sizes from which the databases were derived. Allele frequencies of all SNPs showed significant differences between the 1048 case and control pairs (*P* = 0.0116 for rs863582, *P* = 0.0129 for rs842226, *P* = 0.0091 for rs842225, *P* = 0.0152 for rs2550236, *P* = 0.0036 for rs2688515, *P* = 0.0048 for rs2641773, *P* = 0.0012 for rs3096337, *P* = 0.0016 for rs859769, and *P* = 0.0012 for rs842461).

Significant associations were observed for all nine SNPs (*P = 0.0425* for rs863582, *0.0333* for rs842226, *0.0294* for rs842225, *0.0010* for rs2550236, *0.0149* for rs2688515, *0.0191* for rs 2641773, *0.0058* for rs3096337, *0.0077* for rs859769, and *0.0059* for rs842461 in a additive model) based on the best fit of the AIC. The two tagSNPs (rs863582 and rs842461) remained significant after applying 10,000 permutations (*P* value from empirical distribution of minimal *P* values = 0.0315).

Multivariate logistic regression models showed that after adjusting for confounding factors, compared with wild-type carriers in a dominant model, a significantly increased lung cancer risk was associated with the variant genotypes of rs863582 (T>C) (adjusted OR = 1.39 and 95% CI = 1.02-1.56 for CT/CC genotypes) and rs842461 (A>C) (adjusted OR = 1.25 and 95% CI = 1.05-1.49 for CA/CC genotypes) (Table **2**).

**Table 2:** Genotype frequencies of selected *MUC4* SNPs among cases and controls and their association with lung cancer risk.

Genetic model	Genotype	Cases	Controls	P^a^	Logistic regression	P^c^
		No. (%)	No. (%)		OR (95% CI)^b^	
rs863582						
Additive	TT	478 (45.61)	531 (50.72)	*0.0425*	1.00 (ref.)	
	CT	457 (43.61)	425 (40.59)		1.22 (1.01, 1.47)	*0.0356*
	CC	113 (10.78)	91 (8.69)		1.39 (1.02, 1.89)	*0.0392*
Dominant	CT/CC	570 (54.39)	516 (49.28)	*0.0194*	1.25 (1.05, 1.49)	*0.0132*
rs842226						
Additive	CC	476 (46.21)	532 (51.10)	*0.0333*	1.00 (ref.)	
	TC	443 (43.01)	420 (40.35)		1.14 (0.54, 2.39)	*0.7310*
	TT	111 (10.78)	89 (8.55)		1.43 (1.04, 1.96)	*0.0257*
Dominant	TC/TT	554 (53.79)	509 (48.9)	*0.0260*	1.38 (1.03, 1.85)	*0.0293*
rs842225						
Additive	AA	380 (37.29)	433 (41.96)	*0.0294*	1.00 (ref.)	
	GA	490 (48.09)	481 (46.61)		1.04 (0.77, 1.40)	*0.7959*
	GG	149 (14.62)	118 (11.43)		1.38 (1.03, 1.85)	*0.0301*
Dominant	GA/GG	639 (62.71)	599 (58.04)	*0.0308*	1.10 (1.01, 1.21)	*0.0344*
rs2550236						
Additive	GG	447 (44.26)	511 (49.42)	*0.0010*	1.00 (ref.)	
	AG	454 (44.95)	431 (41.68)		1.18 (0.84, 1.67)	*0.3373*
	AA	109 (10.79)	92 (8.90)		1.42 (1.04, 1.94)	*0.0298*
Dominant	AG/AA	563 (55.74)	523 (50.58)	*0.0194*	1.31 (1.01, 1.69)	*0.0395*
rs2688515						
Additive	AA	418 (40.08)	475 (45.72)	*0.0149*	1.00 (ref.)	
	GA	484 (46.40)	454 (43.69)		1.13 (0.87, 1.47)	*0.3686*
	GG	141 (13.52)	110 (10.59)		1.45 (1.08, 1.95)	*0.0126*
Dominant	GA/GG	625 (59.92)	564 (54.28)	*0.0093*	1.26 (0.99, 1.58)	*0.0521*
rs2641773						
Additive	AA	410 (39.58)	467 (45.04)	*0.0191*	1.00 (ref.)	
	CA	482 (46.52)	456 (43.97)		1.13 (0.86, 1.47)	*0.3812*
	CC	144 (13.90)	114 (10.99)		1.42 (1.06, 1.91)	*0.0176*
Dominant	CA/CC	626 (60.42)	570 (54.96)	*0.0119*	1.24 (0.99, 1.56)	*0.0646*
rs3096337						
Additive	AA	506 (48.28)	574 (54.77)	*0.0058*	1.00 (ref.)	
	GA	437 (41.70)	396 (37.79)		1.22 (0.92, 1.61)	*0.1664*
	GG	105 (10.02)	78 (7.44)		1.58 (1.14, 2.18)	*0.0058*
Dominant	GA/GG	542 (51.72)	474 (45.23)	*0.0030*	1.36 (1.08, 1.71)	*0.0096*
rs859769						
Additive	TT	452 (43.25)	516 (49.28)	*0.0077*	1.00 (ref.)	
	GT	463 (44.31)	433 (41.36)		1.14 (0.90, 1.46)	*0.2769*
	GG	130 (12.44)	98 (9.36)		1.53 (1.13, 2.06)	*0.0057*
Dominant	GT/GG	593 (56.75)	531 (50.72)	*0.0057*	1.26 (1.02, 1.57)	*0.0323*
rs842461						
Additive	AA	502 (48.13)	572 (54.69)	*0.0059*	1.00 (ref.)	
	CA	436 (41.80)	395 (37.76)		1.22 (0.92, 1.62)	*0.1610*
	CC	105 (10.07)	79 (7.55)		1.57 (1.13, 2.16)	*0.0065*
Dominant	CA/CC	541 (51.87)	474 (45.31)	*0.0027*	1.36 (1.08, 1.71)	*0.0099*

^a^ Genotype frequencies in cases and controls were compared using two-sided χ^2^-tests.

^b^ Adjusted for age, sex, pack-years of smoking, and family history of cancer.

^c^ *P* value from unconditional logistic regression analyses.

ref.: reference.

We further assessed the associations of the rs863582 (T>C) and rs842461 (A>C) variant genotypes with lung cancer risk stratified by selected variables and histological types. As shown in Table **3**, compared with the common wild-type homozygous genotype, the adverse effect of rs863582 (T>C) was more evident in smokers (adjusted OR = 1.41 and 95% CI = 1.12-1.79), especially heavy smokers (≥ 20 pack-years, adjusted OR = 1.59 and 95% CI = 1.19-2.13) and in those with severe lung cancer (adjusted OR = 1.34 and 95% CI = 1.04-1.73). Consistent with these results of rs863582 (T>C) genotypes and lung cancer risk analysis, rs842461 (A>C) variant genotype analyses also revealed almost identical change tendencies in different subgroups.

**Table 3:** Stratified analyses between *MUC4* rs863582 and rs842461 genotypes and lung cancer risk.

	MUC4	rs863582				MUC4	rs842461			
	Cases/Controls		OR (95% CI)^a^	*P* ^d^	*P* ^e^	Cases/Controls		OR (95% CI)^a^	*P* ^d^	*P* ^e^
	(1048/1048)					(1048/1048)				
	TT	TC/CC	TC/CC vs. TT			AA	CA/CC	CA/CC vs. AA		
	no.	no.				no.	no.			
Age (years)					*0.6073*					*0.9993*
≤60	241/258	293/269	1.17 (0.92, 1.50)	*0.1959*		241/280	270/240	1.42 (1.03, 1.96)	*0.0335*	
>60	237/273	277/248	1.28 (1.01, 1.64)	*0.0190*		261/266	302/236	1.33 (0.96, 1.85)	*0.0871*	
Sex					*0.9793*					*0.9905*
Male	342/377	402/361	1.24 (1.01, 1.52)	*0.0439*		358/379	407/333	1.40 (1.06, 1.85)	*0.0172*	
Female	136/154	168/156	1.22 (0.89, 1.68)	*0.2183*		144/167	165/143	1.33 (0.88, 2.01)	*0.1730*	
Smoking status^b^					*0.0911*					*0.2004*
Yes	268/287	331/251	1.41 (1.12, 1.79)	*0.0037*		284/307	303/227	1.52 (1.10, 2.10)	*0.0120*	
No	210/244	239/266	1.05 (0.81, 1.36)	*0.7013*		218/239	269/249	1.21 (0.87, 1.68)	*0.2524*	
Packs of year					*0.0429*					*0.0001*
≥20	205/175	252/137	1.59 (1.19, 2.13)	*0.0019*		223/249	184/129	1.70 (1.15, 2.50)	*0.0072*	
<20	63/112	79/114	1.20 (0.79, 1.84)	*0.4006*		61/58	119/98	1.27 (0.72, 2.22)	*0.4074*	
0	210/244	239/266	1.04 (0.81, 1.36)	*0.7013*		218/239	269/249	1.21 (0.87, 1.68)	*0.2524*	
Alcohol use					*0.7634*					*0.0481*
Yes	104/112	125/114	1.14 (0.78, 1.67)	*0.4858*		438/153	513/109	1.62 (1.22, 2.15)	*0.0008*	
No	374/419	445/403	1.24 (1.02, 1.50)	*0.0296*		64/393	59/367	0.99 (0.67, 1.47)	*0.9621*	
Family history of					*0.0785*					*0.9785*
lung cancer										
Yes	21/10	21/20	0.66 (0.22, 1.95)	*0.4480*		45/59	48/50	1.76 (0.82, 3.76)	*0.1472*	
No	457/521	549/497	1.27 (1.06, 1.51)	*0.0084*		457/487	524/426	1.35 (1.06, 1.72)	*0.0148*	
Family history of					*0.3390*					*0.0916*
cancer										
Yes	44/40	60/58	1.06 (0.59, 1.89)	*0.8521*		22/20	12/18	1.18 (0.28, 5.05)	*0.8238*	
No	434/491	510/459	1.25 (1.05, 1.51)	*0.0117*		480/526	560/458	1.42 (1.12, 1.79)	*0.0036*	
Histological types					*-—*					*-—*
Adenocarcinoma	162/531	220/517	1.39 (1.10, 1.77)	*0.0063*		177/572	205/476	1.35 (0.98, 1.85)	*0.0620*	
Squamous cell	170/531	190/517	1.11 (0.90, 1.45)	*0.2905*		182/572	183/476	1.33 (0.96, 1.84)	*0.0841*	
Large cell	17/531	26/517	1.65 (0.88, 3.08)	*0.1195*		20/572	23/476	1.41 (0.61, 3.24)	*0.4203*	
Small cell	66/531	62/517	0.98 (0.68, 1.42)	*0.9137*		63/572	65/476	1.81 (1.09, 2.98)	*0.0209*	
Other carcinomas^c^	52/531	64/517	1.27 (0.86, 1.87)	*0.2222*		53/572	63/476	1.49 (0.88, 2.53)	*0.1430*	
Stage					*-—*					*-—*
I	61/531	87/517	1.34 (0.96, 1.89)	*0.0902*		71/572	83/476	1.48 (0.93, 2.34)	*0.0950*	
II	51/531	43/517	0.88 (0.57, 1.35)	*0.5561*		50/572	44/476	1.31 (0.73, 2.38)	*0.3659*	
III	145/531	185/517	1.34 (1.04, 1.73)	*0.0215*		158/572	172/476	1.34 (0.95, 1.88)	*0.0922*	
IV	215/531	255/517	1.23 (0.99, 1.54)	*0.0605*		223/572	247/476	1.45 (1.08, 1.94)	*0.0127*	

^a^ Adjusted for age, sex, smoking status, alcohol use, and family history of cancer.

^b^ Those who had smoked less than 1 cigarette per day and <1 year in their lifetime were considered nonsmokers; otherwise they were considered smokers (including individuals who had quit for >1 year).

^c^ Mixed-cell or undifferentiated carcinoma.

^d^ *P* value from unconditional logistic regression analyses.

^e^ *P* value of a test of the multiplicative interaction between rs863582 (T>C) and rs842461 (A>C) and selected cancer risk variables, calculated using standard unconditional logistic regression models.

### Association between haplotypes/diplotypes and lung cancer risk

A global score test showed statistically significant differences in haplotype frequency distribution between the cases and controls for blocks 1 (χ^2^ = 43.67, df = 22, *P* value = 0.0039, *P*_simb_ = 0.0021) and 2 (χ^2^ = 12.05, df = 4, *P* value = 0.0169, *P*_simb_ = 0.0107) (Table **4**).

**Table 4:** Association between common *MUC4* haplotypes in each block, and lung cancer risk in overall population and subpopulation stratified by pack-years of smoking.

Haplotype^a^	Overall population						Non	Light	Heavy
							smokers	smokers	smokers
	Case	Control	*P*	*P*_sim_^b^	OR (95%CI)^c^	Global score test	OR (95%CI)^d^	OR (95%CI)^d^	OR (95%CI)^d^
Block 1									
TCAGAA	0.5489	0.6095	*0.0007*	*0.0011*	1.00 (ref.)	χ^2^ = 43.67	1.00 (ref.)	1.00 (ref.)	1.00 (ref.)
CTGAGC	0.2867	0.2618	*0.0059*	*0.0186*	1.25 (1.05, 1.50)	df = 22	1.15 (0.88, 1.51)	0.92 (0.57, 1.49)	1.57 (1.16, 2.13)
TCGGGC	0.0363	0.0370	*0.8965*	*0.8983*	1.08 (0.77, 1.52)	*P* = 0.0039	1.08 (0.65, 1.79)	0.87 (0.59, 2.42)	0.87 (0.52, 1.47)
TCGGAA	0.0203	0.0176	*0.6437*	*0.6474*	1.31 (0.81, 2.11)	*P*_sim_^b^ = 0.0021	1.33 (0.68, 2.58)	1.20 (0.36, 4.00)	1.50 (0.61, 3.68)
TCAAGC	0.0152	0.0105	*0.2515*	*0.2518*	1.62 (0.91, 2.87)		1.35 (0.58, 3.10)	0.67 (0.13, 3.58)	3.42 (0.92, 8.71)
CTGGAA	0.0131	0.0109	*0.5369*	*0.5267*	1.30 (0.73, 2.33)		0.98 (0.40, 2.43)	2.07 (0.60, 7.11)	1.38 (0.50, 3.84)
Block 2						χ^2^ = 12.05			
ATA	0.6516	0.6975	*0.0017*	*0.0019*	1.00 (ref.)	df =4	1.00 (ref.)	1.00 (ref.)	1.00 (ref.)
GGC	0.3082	0.2629	*0.0013*	*0.0015*	1.30 (1.09, 1.55)	*P* = 0.0169	1.18 (0.91, 1.52)	1.20 (0.76, 1.89)	1.60 (1.19, 2.15)
AGA	0.0363	0.0377	*0.8148*	*0.8679*	1.01 (0.73, 1.42)	*P*_sim_^b^ = 0.0107	1.05 (0.64, 1.73)	1.49 (0.54, 4.16)	0.77 (0.47, 1.26)

^a^ Block 1 includes the six common with Minor Haplotype Frequency (MHF) ≥ 0.01 from 27 possible haplotypes; Block 2 includes the three common with MHF ≥ 0.01 from 6 possible haplotypes; Polymorphic bases were in 5'-3' order as listed in Table 1. Loci chosen for block 1, SNP1-6; loci chosen for block2, SNP7-9.

^b^ Generated by 10,000 permutations.

^c^ Adjusted for age, sex, pack-years of smoking, and family history of cancer.

^d^ Adjusted for age, sex, and family history of cancer.

ref.: reference

Logistic regression analyses revealed that lung cancer risk was significantly increased among individuals carrying the haplotype “GGC” (adjusted OR = 1.30 and 95% CI = 1.09-1.55) compared with those carrying the most common haplotype “ATA” in block 2 (Table **4**). Notably, the “GGC” haplotype harbored the rs3096337 G allele and the rs842461 C allele, and these two alleles were both associated with significantly increased lung cancer risk in the single-locus analysis. Furthermore, the stratified analyses revealed that lung cancer risk was further increased among heavy smokers carrying the haplotype “GGC” (adjusted OR = 1.60 and 95% CI = 1.19-2.15). In block 1, both the haplotype and the diplotype analyses revealed an adverse effect of the haplotype “CTGAGC” of rs863582, rs842226, rs2550236, rs842225, rs2688515, and rs2641773 (especially among heavy smokers [adjusted OR = 1.57 and 95% CI = 1.16-2.13]), and this effect is consistent with the single-locus analysis results (Table **S3**).

### Gene-smoking interaction analysis

As summarized in Table **5**, we first classified cumulative smoking dose as a discrete variable (nonsmokers, light smokers, and heavy smokers) to avoid the issue of potential participant misclassification for smoking exposure. The adjusted ORs of the rs863582 TC/CC versus TT genotypes increased significantly as pack-years increased in both the cumulative smoking exposure and the genotype-smoking joint-effects analyses, although the comparisons between light and nonsmokers did not reach statistical significance. When we considered nonsmokers with TT or TC/CC as the reference group in the joint-effects model, heavy smokers with the same genotypes had the greatest risk for lung cancer (OR = 1.73 and 95% CI = 1.27-2.36; OR = 2.50 and 95% CI = 1.85-3.39), suggesting that it is a major risk factor for lung cancer. The genotype-smoking interaction model revealed significant multiplicative interaction between the rs863582 polymorphism (TC/CC versus TT) and trichotomized cumulative smoking dose (*P* < 0.0001). We also observed a consistent and robust result when considering smoking as continuous cumulative smoking dose (square root of pack-years) (*P* < 0.0001). Similar to the results of rs863582, rs842461 exhibited almost identical change tendencies in genotype and cumulative smoking dose analysis. Notably, the adjusted ORs of the rs842461 CA/CC versus AA genotypes increased significantly as pack-years increased in both the cumulative smoking exposure and genotype-smoking joint-effects analyses, although the comparisons between light and nonsmokers also did not reach statistical significance. When taking nonsmokers with AA or CA/CC as the reference group in the joint-effects model, heavy smokers with the same genotypes had the greatest risk for lung cancer (OR = 1.82 and 95% CI = 1.35-2.46; OR = 2.43 and 95% CI = 1.78-3.32), suggesting that it is a major risk factor for lung cancer. The genotype-smoking interaction model revealed significant multiplicative interaction between the rs842461 polymorphism (CA/CC versus AA) and trichotomized cumulative smoking dose (*P* = 0.0001). We also found a consistent and robust result when considering smoking as continuous cumulative smoking dose (square root of pack-years) (*P* = 0.0024).

**Table 5:** Interaction analyses of the *MUC4* rs863582 and rs842461 genotypes and cumulative smoking dose.

Cumulative	Genotype	Case	Control	Stratified	Joint-effect model^b^		Genotype-smoking	
smoking dose		no.	no.	analyses^a^			interaction model	
				OR (95%CI)^c^	OR (95%CI)^d^	OR (95%CI)^e^	*P* ^f^_interaction_	*P* ^g^_interaction_
**rs863582**							*< 0.0001*	*< 0.0001*
Nonsmokers	TT	221	249	1.00 (ref.)	1.00 (ref.)	0.93 (0.72, 1.19)		
Nonsmokers	TC/CC	246	269	1.09 (0.85, 1.41)	1.08 (0.84, 1.39)	1.00 (ref.)		
Light smokers	TT	58	107	1.00 (ref.)	0.73 (0.49, 1.08)	0.67 (0.46, 1.00)		
Light smokers	TC/CC	63	110	1.07 (0.68, 1.68)	0.80 (0.54, 1.17)	0.74 (0.51, 1.08)		
Heavy smokers	TT	211	175	1.00 (ref.)	1.73 (1.27, 2.36)	1.60 (1.19, 2.17)		
Heavy smokers	TC/CC	261	138	1.58 (1.18, 2.11)	2.70 (1.98, 3.68)	2.50 (1.85, 3.39)		
**rs842461**							*0.0001*	*0.0024*
Nonsmokers	AA	218	269	1.00 (ref.)	1.00 (ref.)	0.85 (0.66, 1.09)		
Nonsmokers	CA/CC	239	249	1.18 (0.92, 1.53)	1.18 (0.92, 1.52)	1.00 (ref.)		
Light smokers	AA	61	119	1.00 (ref.)	0.74 (0.51, 1.08)	0.63 (0.43, 1.02)		
Light smokers	CA/CC	58	98	1.14 (0.72, 1.78)	0.87 (0.58, 1.28)	0.73 (0.49, 1.09)		
Heavy smokers	AA	223	184	1.00 (ref.)	1.82 (1.35, 2.46)	1.54 (1.14, 2.08)		
Heavy smokers	CA/CC	249	129	1.61 (1.21, 2.15)	2.87 (2.11, 3.92)	2.43 (1.78, 3.32)		

^a^ Analyses in each stratum of cumulative smoking dose (nonsmokers, light smokers [<20 pack-years] and heavy smokers [≥20 pack-years]).

^b^ Joint effects of rs863582 and rs842461 genotypes and cumulative smoking dose.

^c^ The reference group was comprised of TT and AA genotypes with adjustments for age, sex, and family history of cancer.

^d^ Nonsmokers combined with the TT and AA genotypes were the reference group for analysis with adjustments for age, sex, and family history of cancer.

^e^ Nonsmokers combined with TC/CC and CA/CC were the reference for analysis with adjustments for age, sex, and family history of cancer.

^f^ The genotype-smoking interaction model incorporated specific categories of pack-years (nonsmokers, light smokers, and heavy smokers) as a discrete variable.

^g^ The genotype-smoking interaction model incorporated the square root of pack-years as a continuous variable.

ref.: reference

## Discussion

In the present case-control study, we investigated the effect of multiple common *MUC4* gene variants and their interaction with cigarette exposure on lung cancer risk in a Southern Han Chinese population. We found that nine SNPs (rs863582, rs842226, rs842225, rs2550236, rs2688515, 2641773, rs3096337, rs859769, and rs842461) were significantly associated with lung cancer risk. Consistent with the results of single-locus analysis, the haplotype analyses revealed an adverse effect of the haplotype “GGC” of rs3096337, rs859769, and rs842461 on lung cancer. Both the haplotype and diplotype “CTGAGC” of rs863582, rs842226, rs2550236, rs842225, and rs2688515 had adverse effects on lung cancer risk, which is consistent with the single-locus analysis results. Moreover, we observed a statistically significant interaction for rs863582 and rs842461 with cigarette smoking when tested as either a discrete or continuous variable. These findings support our hypothesis that *MUC4* polymorphisms and their interaction with smoking may contribute to lung cancer etiology. To the best of our knowledge, this is the first study to assess associations for a broad spectrum of genetic variants individually and collectively as haplotypes of the *MUC4* gene and lung cancer risk.

It is biologically plausible that MUC4 may be involved in lung cancer etiology. For example, MUC4 is thought to be a very specific (100%) and sensitive (91.4%) marker in paraffin-embedded lung adenocarcinoma tissue, which could be useful in diagnostic practice in the distinction between malignant mesothelioma and adenocarcinoma [38]. Moreover, *MUC4* overexpression was found to correlate with poor prognosis in small-sized lung adenocarcinomas [39]. Accumulating evidence suggests that MUC4 might also be a potential diagnostic and prognostic marker for other malignancies, such as ductal carcinoma[40–42].

We first of all found that the two tagSNPs, rs842461and rs863582 were associated with lung cancer risk. In the single-locus association analysis, variant genotypes of these two SNPs exhibited a significantly increased risk of lung cancer individually, even after 10,000 permutations. Moreover, we found “GGC” was accounted for a 60% increase in lung cancer risk among heavy smokers, which was consistent with the effect of variant rs842461 genotypes among the same subgroup, suggesting that the adverse effect of “GGC” was indeed driven by the rs842461 C allele and the rs3096337 G allele. These two SNPs were both located in haplotype block 2, which showed a significant and consistent association with lung cancer risk. Notably, block 2 corresponds to intron 1. Existing evidence indicates that the sequence in intron 1 of human genes may play an important role in transcriptional regulation. The role of intron 1 of *MUC4* in gene regulation and the influence of rs842461 are unknown. Although the functional relevance of rs842461 is not yet clear, it is possible that it may increase transcription activator affinity or decrease that of transcription suppressors to the intronic enhancer, thus upregulating *MUC4* expression levels. Further study is warranted to provide experimental evidence in support of this hypothesis.

Our present study also indicates that the effect of rs863582 or rs842461 appears to be strongly modified by cumulative cigarette smoking. Interestingly, the variant genotypes had no effect in nonsmokers or light smokers but were risk factors among heavy smokers compared with their respective wild-type genotypes. For example, heavy smoking (≥20 pack-years) alone only conferred a 1.58-fold increased lung cancer risk for rs863582, but the effect of heavy smoking with the same genotypes was almost 1.73- or 2.50-fold when TT or TC/CC genotype was used as the respective reference in the joint-effects model, indicating a risk-enhancing relationship between smoking and rs863582 genotype variants. Consistent with these results, the rs842461 variant genotype analyses also revealed almost identical change tendencies. The underlying mechanism involved in the interaction between *MUC4* and smoking is not clear. A large number of biologically active molecules, such as cytokines, bacterial products, growth factors, differentiation agents, and other factors (e.g., tobacco smoke) have been found to regulate *MUC4* expression in vitro and/or in vivo in various cell types [43–46]. Therefore, it is likely that smoking might significantly induce *MUC4* expression, and it is possible that the variant allele G of rs3096337 or C of rs842461 also leads to a higher basal expression level of *MUC4* under normal circumstances. As *MUC4* acts as a tumor promoter for lung cancer, the variant allele G of rs3096337 or C of rs842461 exerts a greater adverse effect than that of the wild-type allele among heavy smokers. Therefore, the subjects carrying the rs3096337 variant G or rs842461 variant C may have not have increased lung cancer risk under normal conditions but do have an elevated risk when the G or C allele is in strong LD with a variant allele of another gene (e.g., growth factor genes) that may induce *MUC4* expression in response to heavy smoking. Nevertheless, such speculation requires further support from additional functional studies.

There are three main strengths of this study. First, to the best of our knowledge, no study has evaluated *MUC4* SNPs for associations with lung cancer. Because lung cancer is a multifactorial disease that likely involves multiple SNPs in genes, we assessed a broader spectrum of *MUC4* variants individually as alleles and collectively as haplotypes, which may be more powerful than analyzing a single allele or locus. Second, all lung cancer diagnoses were confirmed by histologic methods, and complete questionnaire data were systematically collected. The adjusted ORs in both stratified and joint-effect analyses for different pack-year categories of smoking were similar in magnitude and direction to the point estimates obtained from fitted ORs of the interaction models. Third, the statistical powers in gene and gene-environment interaction analyses (File **S2**) in this study were sufficient. These consistent results suggest that our findings are not likely to be due to chance. Ultimately, an investigation of a candidate gene requires many SNPs for individual association analysis [47,48], but such testing will increase the false-positive (type I error) rate under nominal significance thresholds (e.g., a = 0.05) except when the selected SNPs are all in high LD with each other. Namely, when background LD exists between SNPs, but they are assumed to be completely independent, then the popular Bonferroni correction would overcorrect for the inflated false-positive rate, resulting in reduced study power [49]. For calculating the significance of SNPs in LD with each other, a permutation test was used to adjust for multiple tests while preserving the correlation structure among linked markers [50–53]. In this way, the false-positive rate for a large number of tests was well controlled in the present study. Despite the strengths and biologic plausibility of the associations observed in the present study, inherent biases may have resulted in spurious findings. Firstly, the lung cancer cases were enrolled from hospitals, and the controls were selected from community health stations and a health examination center, so inherent selection bias cannot be completely excluded. However, we minimized potential confounding factors by matching the controls to the cases on age, sex, and residential area (urban or rural). Secondly, the sample size of the present study may not be large enough either to detect a small effect from very low penetrance SNPs or to identify significant associations of the effect in different strata in subgroup analysis adequately. Thirdly, except for smoking status, other factors such as occupational exposure and nutritional status, which might interact with *MUC4* genotypes or act as potential confounding factors, were not included in our study. Possible interactions between *MUC4* genotypes and these risk factors should be thoroughly investigated in future work. Ultimately, the functional relevance of rs863582 and rs842461 are unknown and should be assessed.

In conclusion, our study provides evidence that *MUC4* polymorphisms and their interactions with smoking status may contribute to lung cancer etiology in a Chinese population. Moreover, we also demonstrated that genetic susceptibility, coupled with a modifiable lifestyle factor (i.e., smoking status), and appeared to confer a significantly higher risk of lung cancer than either factor alone. These findings need to be substantiated by larger-scale studies in different ethnic populations.

## Supporting Information

Figure S1 **MUC4 rs863582 T>C, rs842226 C>T, rs842225 A>G, rs2550236 G>A, rs2688515 A>G, rs2641773 A>C, rs3096337 A>G, rs859769 T>G, and rs842461 A>C; genotyping by Taqman assays.**
(TIF)

Table S1 **Linkage disequilibrium (D´ and r^2^) between SNPs in *MUC4* in control subjects.**
(DOC)

Table S2 **The primers and probes for Taqman-PCR on 9 genotyped SNPs of *MUC4* gene.**
(DOC)

Table S3 **Main effects of common diplotypes on lung cancer risk.**
(DOC)

Table S4 **Frequency distributions of selected variables in lung cancer and cancer-free control subjects.**
(DOC)

File S1 **Chromosome position of 296 selected SNPs of *MUC4* gene, their MAF values and function from dbSNP (accessed 9/9/2012) and/or Hapmap_CHB_Rel28 databases.**
(XLSX)

File S2 **Table A: Statistical powers in gene analysis; Table B: Statistical powers in gene and environment interaction analysis.**
(XLSX)
