# Supplementary figures and images for: Genetic Variants in MUC4 Gene Are Associated with Lung Cancer Risk in a Chinese Population
Source: PLoS One. 2013 Oct 21;8(10):e77723. doi: 10.1371/journal.pone.0077723 (PMC3804582; doi:10.1371/journal.pone.0077723)

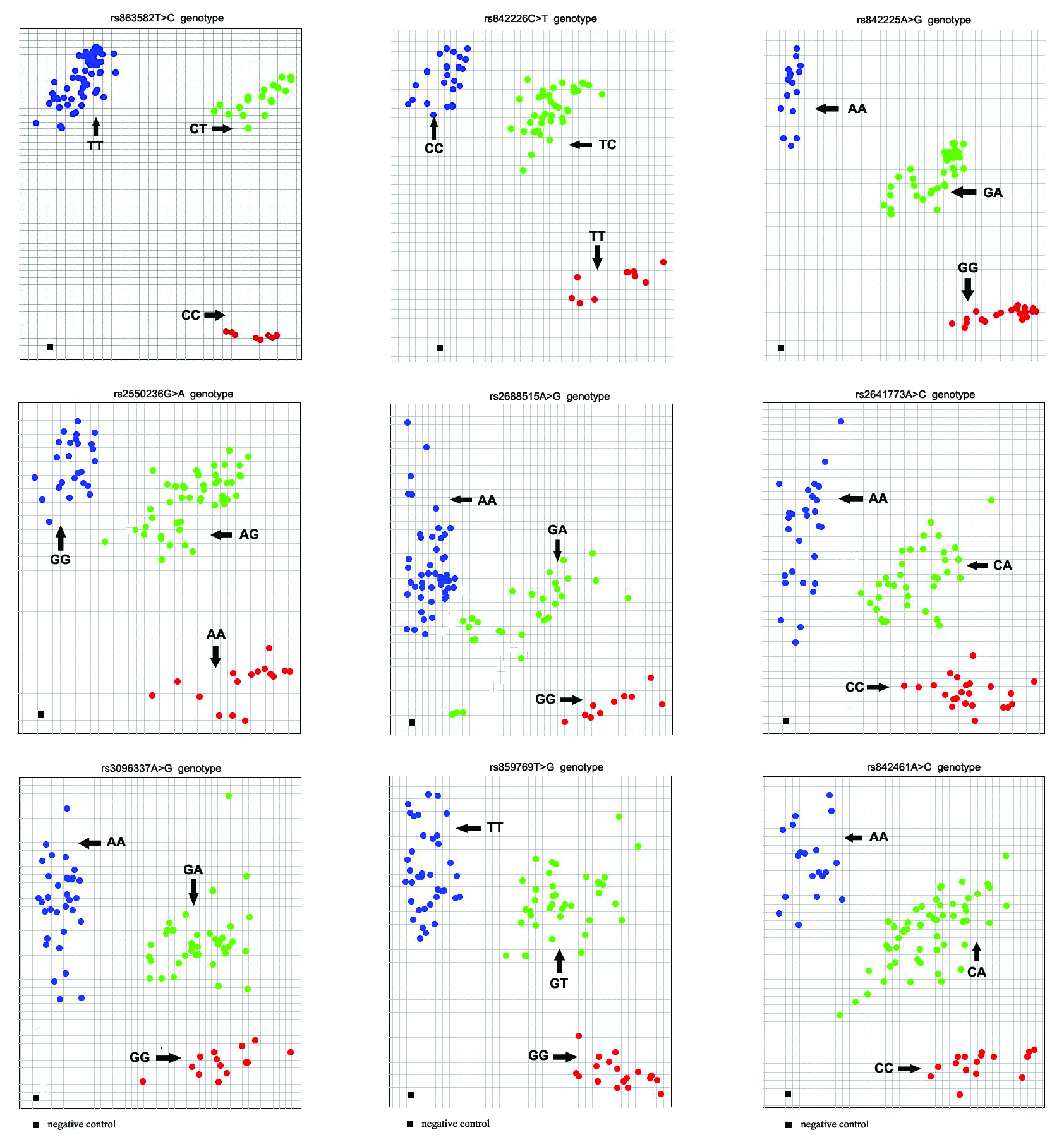

Supplement: Figure S1 — MUC4 rs863582 T>C, rs842226 C>T, rs842225 A>G, rs2550236 G>A, rs2688515 A>G, rs2641773 A>C, rs3096337 A>G, rs859769 T>G, and rs842461 A>C; genotyping by Taqman assays. (TIF) [file pone.0077723.s001.tif]
