# Supplementary material for: Genetic Variants in MUC4 Gene Are Associated with Lung Cancer Risk in a Chinese Population
Source: PLoS One. 2013 Oct 21;8(10):e77723. doi: 10.1371/journal.pone.0077723 (PMC3804582; doi:10.1371/journal.pone.0077723)
Supplement: Table S1 — Linkage disequilibrium (D´ and r2) between SNPs in MUC4 in control subjects. (DOC) [file pone.0077723.s002.doc]

**Table S1.** Linkage disequilibrium (D´ and r*^2)^* between SNPs in *MUC4* in control subjects

*D*’ values are given above the diagonal; *r^2^* values are given below the diagonal.

| SNPs | rs863582 | rs842226 | rs842225 | rs2550236 | rs2688515 | rs2641773 | rs3096337 | rs859769 | rs842461 |
| --- | --- | --- | --- | --- | --- | --- | --- | --- | --- |
| rs863582 | — | 0.995 | 0.986 | 0.925 | 0.898 | 0.897 | 0.909 | 0.787 | 0.906 |
| rs842226 | 0.972 | — | 0.983 | 0.926 | 0.893 | 0.894 | 0.893 | 0.788 | 0.890 |
| rs842225 | 0.746 | 0.727 | — | 0.904 | 0.935 | 0.919 | 0.916 | 0.929 | 0.913 |
| rs2550236 | 0.837 | 0.823 | 0.694 | — | 0.933 | 0.933 | 0.981 | 0.827 | 0.978 |
| rs2688515 | 0.686 | 0.666 | 0.789 | 0.764 | — | 0.995 | 0.988 | 0.990 | 0.986 |
| rs2641773 | 0.673 | 0.657 | 0.776 | 0.752 | 0.976 | — | 0.988 | 0.990 | 0.985 |
| rs3096337 | 0.726 | 0.715 | 0.571 | 0.827 | 0.732 | 0.721 | — | 1.000 | 1.000 |
| rs859769 | 0.583 | 0.574 | 0.707 | 0.664 | 0.887 | 0.874 | 0.828 | — | 1.000 |
| rs842461 | 0.726 | 0.714 | 0.570 | 0.824 | 0.729 | 0.718 | 0.998 | 0.825 | — |
