# Supplementary material for: Genetic Variants in MUC4 Gene Are Associated with Lung Cancer Risk in a Chinese Population
Source: PLoS One. 2013 Oct 21;8(10):e77723. doi: 10.1371/journal.pone.0077723 (PMC3804582; doi:10.1371/journal.pone.0077723)
Supplement: Table S2 — The primers and probes for Taqman-PCR on 9 genotyped SNPs of MUC4 gene. (DOC) [file pone.0077723.s003.doc]

**Table S2. The primers and probes for Taqman-PCR on 9 genotyped SNPs of *MUC4* gene**

| NCBI | Primers^a^ | Probes |
| --- | --- | --- |
| SNP ID |  |  |
| rs863582 |  |  |
|  | F:CCTGAGTCAACAGGCCGTATC | P1:FAM-CCGTTTAAATGATAA**T**GCGTGT |
|  | R:TGTCAGGCACTGGACTAAATGC | P2:HEX-CGTTTAAATGATAA**C**GCGT |
| rs842226 |  |  |
|  | F:CATACCTCAGTCACAGGCAAAGTG | P1:FAM-ACAACTGC**C**GAGTGG |
|  | R: CTGGGTGTGCAGGATTCTACAG | P2:HEX-CACAACTGC**T**GAGTGG |
| rs842225 |  |  |
|  | F: ATGGTGTAAGGTGCGGGCTAT | P1:FAM-AGGCAAG**T**TGTCACGG |
|  | R: TCTCTGCCTCCGTCCTTCCT | P2:HEX-AGGCAAG**C**TGTCACGG |
| rs2550236 |  |  |
|  | F:TCAGCAATATGGAAGAAGAGATAAGC | P1:FAM-CAAAACCAGCC**G**ATG |
|  | R:TCTCACCTCTGCATTAGAAAACATTT | P2:HEX-CAAAACCAGCC**A**ATG |
| rs2688515 |  |  |
|  | F:CACAGGATTTCTTACCTACAAATAACCTAC | P1:FAM-TAAACAGCT**A**AATAATCAATCT |
|  | R:CTACAGTTATTGCATTTGTCTTTCACC | P2:HEX-TAAACAGCT**G**AATAATCAATCT |
| rs2641773 |  |  |
|  | F: GCACGGCTTTGGTTCTTTCA | P1:FAM-AAGTAGAAAATGGAG**T**GTCA |
|  | R: CCTAAAGAGTTGATATTGGAGAGAAACA | P2:HEX-AAGTAGAAAATGGA**G**GGTC |
| rs3096337 |  |  |
|  | F:AAACAGACGTGGCCCAGTCT | P1:FAM-TAATTAC**A**GAAATGTTCTCA |
|  | R:CCATTCGAAATGCAGCTCTCA | P2:HEX-TAATTAC**G**GAAATGTTCTCA |
| rs859769 |  |  |
|  | F:TAGGCTGAGTTCCCGCCC | P1:FAM-TGGCACCTCAT**A**GAC |
|  | R:GGGACAGAGAGAAACTCTGTCTCAA | P2:HEX-TTGGCACCTCAT**C**GA |
| rs842461 |  |  |
|  | F:CTCCAGGACACTCAAAAGGAAAG | P1:FAM-CCAGGAA**A**ATCAGCCAA |
|  | R:GTGCACCTTGCTGGTCGTT | P2:HEX-CAGGAA**C**ATCAGCCAA |

^a^ Primers :F and R mean Forward and Reverse ,respectively.
