# Supplementary material for: Genetic Variants in MUC4 Gene Are Associated with Lung Cancer Risk in a Chinese Population
Source: PLoS One. 2013 Oct 21;8(10):e77723. doi: 10.1371/journal.pone.0077723 (PMC3804582; doi:10.1371/journal.pone.0077723)
Supplement: Table S3 — Main effects of common diplotypes on lung cancer risk. (DOC) [file pone.0077723.s004.doc]

**Table S3. Main effects of common diplotypes on lung cancer risk**

| Haplotypes | 0copy ^a^ | 1~2copies |  |  |  |
| --- | --- | --- | --- | --- | --- |
|  | Case/Control | Case/Control |  | OR_crude_(95%CI) | OR_adjusted_(95%CI)^b^ |
| Block1 |  |  |  |  |  |
| TCAGAA | 477/404 | 571/644 |  | 0.75(0.63,0.90) | 0.73(0.61,0.88) |
| CTGAGC | 738/782 | 310/266 |  | **1.25(1.02,1.52)** | **1.22(1.01,1.48)** |
| TCGGGC | 1012/1007 | 36/41 |  | 0.87(0.54,1.41) | 0.85(0.54,1.35) |
| TCGGAA | 1028/1027 | 20/21 |  | 0.95(0.49,1.84) | 1.01(0.54,1.91) |
| TCAAGC | 1032/1032 | 16/16 |  | 1.00(0.47,2.11) | 1.01(0.49,2.09) |
| CTGGAA | 1035/1035 | 13/13 |  | 1.00(0.43,2.30) | 0.96(0.43,2.11) |
| Block2 |  |  |  |  |  |
| ATA | 315/331 | 733/717 |  | 1.07(0.89,1.30) | 1.06(0.68,1.66) |
| GGC | 773/762 | 275/286 |  | 0.95(0.78,1.16) | 0.91(0.75,1.10) |
| AGA | 1008/1005 | 40/43 |  | 0.93(0.58,1.47) | 0.89(0.69,1.12) |

^a^ Defined 0 copy as references of each common haplotype (MAF>0.01).

^b^ Adjusted for age,gender,pack-years of smoking and family history of cancer.
