# Supplementary material for: Genetic Variants in MUC4 Gene Are Associated with Lung Cancer Risk in a Chinese Population
Source: PLoS One. 2013 Oct 21;8(10):e77723. doi: 10.1371/journal.pone.0077723 (PMC3804582; doi:10.1371/journal.pone.0077723)
Supplement: Table S4 — Frequency distributions of selected variables in lung cancer and cancer-free control subjects. (DOC) [file pone.0077723.s005.doc]

**Table S4.** Frequency distributions of selected variables in LC patients and cancer-free controls

| Variables | Patients(n=1048) | |  | Controls(n=1048) | |  | *P^a^* |
| --- | --- | --- | --- | --- | --- | --- | --- |
|  | *N* | % |  | *N* | % |  |  |
| Age (years) |  |  |  |  |  |  | 0.7597 |
| ≤60 | 534 | 50.95 |  | 527 | 50.29 |  |  |
| >60 | 514 | 49.05 |  | 521 | 49.71 |  |  |
| Sex |  |  |  |  |  |  | 0.7734 |
| Male | 744 | 70.99 |  | 738 | 70.42 |  |  |
| Female | 304 | 29.01 |  | 310 | 29.58 |  |  |
| Smoking status |  |  |  |  |  |  | **0.0075** |
| Yes | 599 | 57.15 |  | 538 | 51.34 |  |  |
| No | 449 | 42.84 |  | 510 | 48.66 |  |  |
| Packs of year |  |  |  |  |  |  | **<.0001** |
| ≥20 | 457 | 43.61 |  | 312 | 29.77 |  |  |
| <20 | 142 | 13.55 |  | 226 | 21.56 |  |  |
| 0 | 449 | 42.84 |  | 510 | 48.66 |  |  |
| Drinking status |  |  |  |  |  |  | 0.8737 |
| Yas | 229 | 21.85 |  | 226 | 21.57 |  |  |
| No | 819 | 78.15 |  | 822 | 78.44 |  |  |
| Family history of cancer |  |  |  |  |  |  | 0.6570 |
| Yes | 104 | 9.92 |  | 98 | 9.35 |  |  |
| No | 944 | 90.08 |  | 950 | 90.65 |  |  |
| Family history of lung cancer |  |  |  |  |  |  | 0.1501 |
| Yes | 42 | 4.01 |  | 30 | 2.86 |  |  |
| No | 1006 | 95.99 |  | 1018 | 97.14 |  |  |
| Histological types |  |  |  |  |  |  |  |
| Adenocarcinoma | 384 | 36.64 |  |  |  |  |  |
| Squamous cell | 368 | 35.11 |  |  |  |  |  |
| Large cell | 37 | 3.53 |  |  |  |  |  |
| Small cell | 121 | 11.55 |  |  |  |  |  |
| Other carcinomas | 138 | 13.17 |  |  |  |  |  |
| Stages |  |  |  |  |  |  |  |
| I | 156 | 14.89 |  |  |  |  |  |
| II | 96 | 9.16 |  |  |  |  |  |
| III | 318 | 30.34 |  |  |  |  |  |
| IV | 478 | 45.61 |  |  |  |  |  |

*^a^P* values for a two-sided χ^2^ test.
